# Calcium channel blockade with nimodipine reverses MRI evidence of cerebral oedema following acute hypoxia

**DOI:** 10.1177/0271678X17726624

**Published:** 2017-08-31

**Authors:** Matthew J Rowland, Martyn Ezra, Anderson Winkler, Payashi Garry, Catherine Lamb, Michael Kelly, Thomas W Okell, Jon Westbrook, Richard G Wise, Gwenaëlle Douaud, Kyle TS Pattinson

**Affiliations:** 1 Nuffield Division of Anaesthetics, Nuffield Department of Clinical Neurosciences, University of Oxford, UK; 2 FMRIB, Wellcome Centre for Integrative Neuroimaging, Nuffield Department of Clinical Neurosciences, University of Oxford, UK; 3 Neurosciences Intensive Care Unit, Oxford University Hospitals NHS Trust, Oxford, UK; 4 Preclinical Imaging Facility, Core Biotechnology Services, University of Leicester, Leicester, UK; 5 Cardiff University Brain Research Imaging Centre, School of Psychology, Cardiff University, Cardiff, UK

**Keywords:** Apparent diffusion coefficient, calcium, hypoxia, MRI, nimodipine

## Abstract

Acute cerebral hypoxia causes rapid calcium shifts leading to neuronal damage and death. Calcium channel antagonists improve outcomes in some clinical conditions, but mechanisms remain unclear. In 18 healthy participants we: (i) quantified with multiparametric MRI the effect of hypoxia on the thalamus, a region particularly sensitive to hypoxia, and on the whole brain in general; (ii) investigated how calcium channel antagonism with the drug nimodipine affects the brain response to hypoxia. Hypoxia resulted in a significant decrease in apparent diffusion coefficient (ADC), a measure particularly sensitive to cell swelling, in a widespread network of regions across the brain, and the thalamus in particular. In hypoxia, nimodipine significantly increased ADC in the same brain regions, normalizing ADC towards normoxia baseline. There was positive correlation between blood nimodipine levels and ADC change. In the thalamus, there was a significant decrease in the amplitude of low frequency fluctuations (ALFF) in resting state functional MRI and an apparent increase of grey matter volume in hypoxia, with the ALFF partially normalized towards normoxia baseline with nimodipine. This study provides further evidence that the brain response to acute hypoxia is mediated by calcium, and importantly that manipulation of intracellular calcium flux following hypoxia may reduce cerebral cytotoxic oedema

## Introduction

Many important cellular processes are mediated by calcium. The movement and storage of calcium in cells are subject to tight regulatory control, primarily through the action of voltage gated calcium channels (L-type channels).^1^ In neurons, these calcium channels are the principal source for calcium entry and membrane depolarization after energy failure.^2,3^ Drugs, such as nimodipine, that block these calcium channels may reduce calcium influx into neurons and play a key role in limiting neuronal cellular damage due to oxygen deprivation (cerebral hypoxia).^2^

Nimodipine is an L-type calcium channel blocker characterized by a highly selective action on cerebral blood vessels and a high affinity for receptors in the brain.^4,5^ This preferential cerebral action of the drug has been explained by high transfer across the blood–brain barrier compared with other calcium channel blockers.^6^ Nimodipine has been shown to improve outcomes after aneurysmal subarachnoid haemorrhage, but this effect does not extend to other types of acute brain injury such as trauma and stroke where there is restriction in blood supply (ischaemia).^7^ The precise mechanisms behind this beneficial effect remain unclear, but is likely to be related to blocking calcium influx after tissue ischaemia at a neuronal level.^8^

In both focal and global cerebral ischaemia, there is substantial evidence of variability in the tolerance of different brain regions to hypoxic-ischaemic damage. Sub-cortical structures including the thalamus, the hypothalamus, basal ganglia and cerebellum are particularly sensitive to hypoxic-ischaemic injury,^9–11^ with the thalamus in particular showing early vulnerability in the acute phase.^12^ Poor functional recovery, (specifically in memory) has also been linked to damage in the thalamus in animal studies,^13^ as well as cognitive dysfunction in patients surviving cardiac arrest and traumatic brain injury.^14,15^ The thalamus is therefore a particularly interesting brain structure in which to investigate the role of calcium in mediating cerebral injury following hypoxia, especially as thalamic voltage dependent calcium channels are likely to play a key role in higher-level cortical functions.^16^

Exposure of healthy volunteers to experimental hypoxia potentially offers a model of in vivo reversible brain injury. Heterogeneous changes in diffusion-weighted imaging metrics (e.g. apparent diffusion coefficient (ADC) – an MRI marker of cerebral oedema), cerebral blood flow (CBF) and cerebral volume have all been previously reported after acute hypoxia.^17–19^ There has also been recent interest in the significance of the amplitude of low frequency fluctuations (ALFF) in resting state functional MRI in health and in disease. Changes in ALFF have been observed following traumatic brain injury^20^ and in Parkinson’s disease.^21^ Low-frequency oscillations in calcium (known as a biomarker of cellular oscillations) show frequencies similar to those of deoxyhemoglobin (the main contributor to the BOLD MRI signal) and precede them by 5–6 s.^22^ Changes in ALFF due to cellular calcium flux might therefore represent a biomarker of acute cerebral tissue hypoxia and be influenced by calcium channel blockade with nimodipine.

The purpose of this study was two-fold: first, to test the hypothesis that acute hypoxia would have a significant effect on the thalamus, and second, that this hypoxic effect would be attenuated by the administration of nimodipine. To test these hypotheses, the objectives of this study were to quantify changes in ADC, grey matter (GM) volume, CBF and ALFF associated with acute hypoxia using MRI in healthy humans. We then aimed to investigate the impact of calcium channel blockade with nimodipine on these changes, to offer mechanistic insights into therapeutic calcium channel blockade clinically in patients.

## Materials and methods

The study was conducted in accordance with the Helsinki Declaration as revised in 2008 and was approved by the local UK National Research Ethics Service Committee (NRES Committee South Central – Berkshire: 11/SC/0519). Informed written consent was obtained from all participants. Twenty right handed, healthy volunteers (11 males and 9 females, mean age 28 ± 8 years) with no regular medication were recruited. Specific exclusion criteria included routine contraindications to MRI scanning, history of smoking and recent air travel or exposure to high altitude within the last month.

### Experimental design

**Figure 1.**
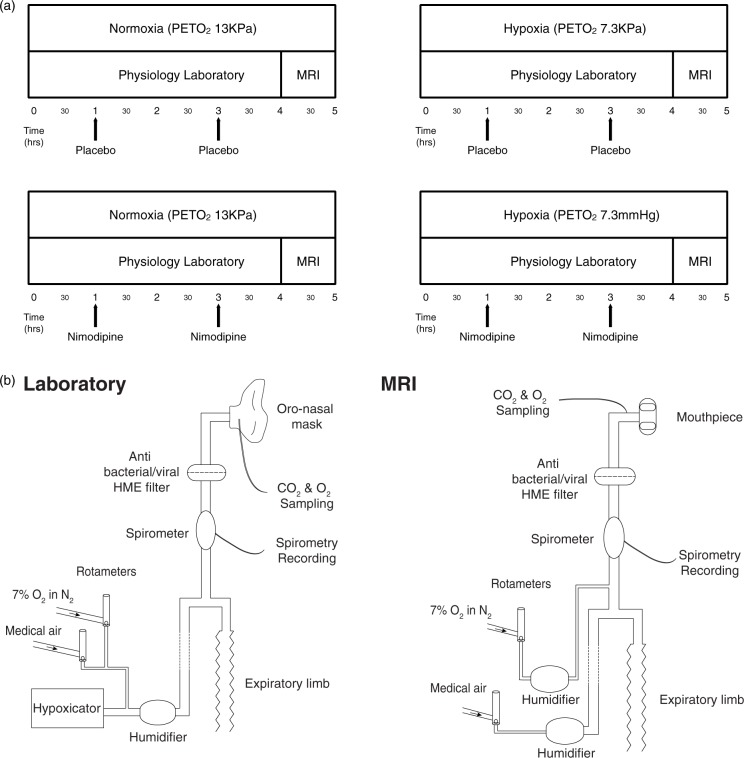
(a) Study protocol. (b) Schematic diagram of the breathing apparatus used to maintain hypoxic conditions in both the laboratory and MRI sessions.

Participants attended four separate experimental sessions (a minimum of one week apart). Each session lasted approximately 5 h in total. The experiment was a factorial design with the order of each session randomised for each subject. The experimental conditions are shown in Figure 1(a) and were as follows:

1. Poikilocapnic normoxia (“normoxia”)/Placebo
2. Poikilocapnic normoxia (“normoxia”)/Nimodipine
3. Poikilocapnic hypoxia (“hypoxia”)/Placebo
4. Poikilocapnic hypoxia (“hypoxia”)/Nimodipine

All conditions were normobaric. Drug/placebo conditions were double blinded for each session with doses prepared by an individual independent of the research group. Participants were blinded to gas mixture in all sessions. The researchers running each experimental session were not blinded to the gas mixture. First level MRI analysis was undertaken blinded to both normoxia/hypoxia and drug/placebo conditions. Un-blinding was only performed once all first level MRI pre-processing and analysis had been completed.

### Experimental procedure

The study protocol consisted of a continuous 4-h session in a physiology laboratory breathing either room air, with the usual fraction of inspired oxygen (FiO_2_) of 21%, or a hypoxic gas mixture with the partial pressure of end-tidal oxygen (PetO_2_) maintained at 7.3 KPa, followed by an MRI scan with PetO_2_ maintained at the same level as during the preceding 4 h. Depending on the session protocol (as per Figure 1(a)), participants received either nimodipine 30 mg or placebo at both 1 h and 3 h into the protocol depending on the experimental condition.

### Acute hypoxia

Modulation of inspired O_2_ content during both the laboratory and MRI sessions was achieved using a custom-built breathing apparatus (Figure 1(b)). During the laboratory hypoxia condition, subjects inhaled gas from a hypoxicator unit (HYP-123 Hypoxicator generator, Hypoxico Inc) with an FiO_2_ of 12% (equivalent to an altitude of 4400 m) via a tight-fitting silicone facemask (Hans Rudolph, 7540 series V2, Kansas City, MO, USA) to maintain an PetO_2_ of 7.3KPa. Additional 7% O_2_ in nitrogen from a cylinder was added as required to ensure tight control of PetO_2_ for each participant. During the normoxia condition, subjects received medical air at the same rate through the same system.

The partial pressure of end-tidal carbon dioxide (PetCO_2_) was not experimentally controlled during the laboratory and MRI sessions to replicate normal cerebral physiological responses to hypoxia. Continuous physiologic recordings were obtained for PetCO_2_ and PetO_2_ (Gas Analyzer, AD Instruments, UK). Oxygen saturations (SpO_2_), heart rate and non-invasive blood pressure (NIBP) were measured every 5 min (Dinamap Procare 300, GE Healthcare). For each subject, the hypoxicator was turned on during all four sessions to ensure the background noise was the same and that subjects remained blinded to the gas mixture condition. During the MRI session, participants used a similar breathing apparatus and a diving mouth-piece (Scubapro Ltd, UK) to maintain the same PetO_2_ as used in the laboratory session for the duration of the MRI scan.

### Study drug

Nimodipine and placebo doses were produced by Ipswich NHS Pharmacy Manufacturing Unit. Capsules were matched in colour, size and shape to ensure both participants and investigators remained blinded to the experimental condition. The dosing and oral route were chosen to replicate standard clinical practice in patients with subarachnoid haemorrhage and timed to aim for maximum serum concentrations of the drug at the start of the MRI scanning session (based on data from Hernández-Hernández R. et al.^23^). Although it was difficult to ensure peak concentrations of nimodipine at the same timepoint in each session, blood samples were taken from each participant at the 4 h mark in each session and any delay between blood sampling and MRI scanning was the same for each session. Serum nimodipine levels were then measured using customised mass spectroscopy (Sequani Ltd).

### MRI scanning

A Siemens MAGNETOM 3T Verio scanner (Siemens Healthcare, Erlangen, Germany) situated immediately adjacent to the physiology laboratory was used for all scanning sessions. At the end of the 4-h laboratory session, participants were moved immediately to the scanner room and placed directly onto the breathing system. Disconnection time was kept to an absolute minimum. Heart rate, SpO_2_) and NIBP were continuously monitored during MRI scans using an MRI-compatible physiological monitoring system (3150/3155 MRI patient monitor, In Vivo Research). Tidal O_2_ and CO_2_ were monitored using a gas analyzer (Gas Analyzer, AD Instruments, NZ).

The scanning protocol included:

- **T1-weighted MPRAGE structural to measure changes in GM volume**: 2 min 22 s, whole brain; 1.5 × 1.5 × 1.5 mm resolution; TR 1780 ms; TI 900 ms; TE 4.4 ms.
- **Diffusion-weighted (DWI) echo planar, spin echo sequence to measure changes in ADC**: 1 min; TR 5300 ms; TE 91 ms; b values 0 and 1000 s/mm^2^, voxel size 1.8 × 1.8 × 5 mm; 25 slices, isotropic encoding.
- **Time-of-flight MR neck angiogram, to facilitate vessel labeling**:1.5 min; 20 slices; 1.2 × 0.8 × .1.3 mm; TR 26 ms; TE 3.43 ms; flip angle 18°.
- **Multi-inversion time (TI), vessel-encoded pseudo-continuous arterial spin labeling (VEPCASL) perfusion-weighted imaging to measure changes in CBF** (as per Okell et al.^24^): 5 min 55 s, 3.4 × 3.4 × 4.5 mm, field of view 220 mm; TR 4080 ms; TE 14 ms, echo planar imaging readout, 24 slices, background suppression with multiple post-labeling delays (tag duration 1.4 s with six delays: 0.25 s, 0.5 s, 0.75 s, 1 s, 1.25 s, 1.5 s) along with calibration scans acquired with both head and body coils for signal reception (24 s each, TR 6000 ms)
- **Phase contrast scan to quantify flow velocities through carotid/vertebral vessels**: 1 min 27 s, 1.9 × 1.9 × 5 mm, TR 58.75 ms, TE 6.06 ms, flip angle 15°, VENC 100 cm/s.
- **Resting state gradient-echo, echo-planar blood oxygen dependent level (BOLD) imaging sequence to measure low-frequency fluctuations in the BOLD signal**: 6 min; TR 3000 ms; TE 40 ms; flip angle 90°; FOV 240 mm; voxel size 3.0 × 3.0 × 3.0 mm, 40 slices
- **B0 fieldmap images** to aid registration by correcting for echo-planar imaging distortion artifacts.

The scanning protocol was ordered as presented above and was conducted in the same order for each experimental session. To quantify any potential change in velocity through the carotid and vertebral arteries due to either hypoxia or nimodipine, phase contrast images were acquired. This technique utilizes the phase of an image to encode the velocity of flowing spins and has been validated for angiogram and quantitative flow measurements.^25^

### Post-processing of MRI data

All imaging analyses were carried out using FSL Version 5.0 (FMRIB Software Library, Functional Magnetic Resonance Imaging of the Brain Centre, Department of Clinical Neurosciences, University of Oxford, Oxford, UK, http://www.fmrib.ox.ac.uk^26^) unless otherwise specified.

#### Volumetric structural data analysis

An optimized voxel-based morphometry (VBM) analysis was undertaken on the T1-weighted images to identify regional changes in the thalamus and whole brain GM volume (FSL-VBM: http://fsl.fmrib.ox.ac.uk/fsl/fslwiki/FSLVBM^27^). All images were processed following the same protocol: first, structural images from all four experimental conditions, in all subjects, were brain-extracted and GM-segmented before being registered to the MNI152 standard space using non-linear registration. The resulting images were averaged and flipped along the x-axis to create a left-right symmetric, study-specific GM template. Second, all native GM images were non-linearly registered to this study-specific template and “modulated” to correct for local expansion (or contraction) due to the non-linear component of the spatial transformation. The modulated GM images were then smoothed with an isotropic Gaussian kernel with a sigma of 3 mm. A thresholded (at 35%) binarised probabilistic mask (Harvard-Oxford, FSL) of the thalamus in MNI152 standard space was used to obtain values for GM volume of both the thalami.

We also investigated voxel-wise changes in volume over the whole brain GM, using permutation-based non-parametric testing (5000 permutations), correcting for multiple comparisons across space (see ‘Statistical analysis’ section below for further details).

#### DWI data analysis

ADC maps obtained from the scanner were brain extracted using the FSL tool BET^28^ and registration of the B0 image to the native T1-weighted image was estimated for each subject with boundary based recognition.^29–31^ Next, we combined this within-subject registration with the non-linear registration of each subject’s GM-segmented image to the VBM template. At the end of this registration process, ADC maps were therefore in the study-specific VBM template space to ensure optimal group registration. The same probabilistic mask of the thalamus used in the VBM analysis was applied to maps from each condition to obtain average values for each participant. Finally, voxel-wise statistical test was applied using permutation-based non-parametric testing to look for changes in GM ADC across the whole brain between the conditions of interest.

#### Perfusion-weighted data analysis

All related data processing steps essential for quantification of CBF including tissue segmentation, estimation of equilibrium magnetization of blood (M_0_b) from the mean CSF (cerebrospinal fluid) magnetization (M_0_csf) within a ventricle mask, and generation of absolute CBF in physiologic units (ml blood/100 g tissue/minute) were completed using FSL tools and MATLAB (MATLAB R2015a, The MathWorks Inc., Natick, MA, 2000) as per Okell et al.^24^

Firstly, head motion was corrected using multi-resolution rigid body co-registration of volumes, as implemented by MCFLIRT. Then multi-TI VEPCASL data was processed using a non-linear fit to the general arterial spin labeling (ASL) kinetic model for all voxels within a whole brain mask, accounting for macrovascular signal, to quantify CBF.^24,32^ Similarly to the ADC analysis, total perfusion maps were registered to the study-specific VBM template space by combining the spatial transformation of each calibration body EPI image to the same subject’s T1-weighted image with the corresponding VBM warpfield. Again, the probabilistic mask of the thalamus was used to obtain average values for thalamic CBF.

Phase contrast MRI was used to assess the blood velocity in the carotid and vertebral arteries at the location of the PCASL labelling plane.^25^ The phase contrast MRI acquisition was cardiac-gated using a pulse oximeter. Vessel ROIs were manually created and applied to the cardiac gated velocity maps to provide plots of blood velocity over the cardiac cycle. Average velocity was then computed and compared to a simulated plot of inversion efficiency versus velocity. Average vessel velocities in the range of 15 cm/s to 45 cm/s result in an inversion efficiency of approximately 0.9,^33^ and were in this range for all subjects. As a result, the effects of blood velocity at the labelling plane on inversion efficiency were likely to be consistent across all subjects.

#### Amplitude of low frequency fluctuations analysis

Head motion was corrected using multi-resolution rigid body co-registration of volumes, as implemented by MCFLIRT. Brain extraction was carried out for motion corrected BOLD volumes as implemented in BET. This procedure was verified with visual inspection of the extraction result for each data set acquired.

Data denoising was performed using an independent component analysis (ICA) approach which was used to decompose FMRI data into different spatial and temporal components using FSL’s MELODIC (Multivariate Exploratory Linear Optimised Decomposition into Independent Components^34^). The noise components were manually identified based on spatial location of signal, time course and signal frequency.^35^ The FSL tool FIX^36,37^ was then used as follows:

1. To regress the full space of the motion confounds obtained from MCFLIRT from both the data and from all the ICA component time-series.
2. To estimate the contribution of both “signal” and “noise” components as a means of identifying the noise specific variance.
3. To remove the unique contribution of the components identified above as “noise” from the data.

Additional data denoising in 13 subjects was also performed using a combination of physiological noise modelling integrated in FEAT (RETROICOR^38,39^) and ICA (described in ^40^) to assess the impact of measured changes in respiratory and heart rate on ALFF between sessions. We were unable to perform this correction on all datasets as five subjects had intermittent short (5–10 s) interruptions in continuous physiological traces during scanning which rendered analysis with RETROICOR impossible, so these five subjects were excluded from this supplementary analysis. Results and figures from this analysis are presented in the Supplementary Material.

We again registered the fMRI volumes to the VBM study-specific template space. The resulting images were smoothed with 6 mm full width at half maximum (FWHM) Gaussian kernel.

Calculation of ALFF was then performed with customised MATLAB (Mathworks Inc.) scripts using the Chronux Toolbox (www.chronux.org). The power of the low frequency fluctuations in the frequency range 0.01–0.1 Hz was measured using a multi-taper spectral estimation^41^ using discrete prolate spheroidal (Slepian) sequences with five tapers, Fourier transform algorithm for the resting-state time series for each voxel. The power between the 0.01 and 0.1 Hz band was divided by the total power of the resolved frequencies from 0 Hz to the Nyquist frequency to calculate normalised spectral power for that low frequency band. This was performed to look specifically at changes within this low power band. We used the same probabilistic mask of the thalamus to obtain average values for ALFF in the thalamus region.

### Statistical analysis

The contrasts of interest between conditions were:

1. Effect of hypoxia on brain: Normoxia/Placebo v Hypoxia/Placebo.
2. Effect of nimodipine in normoxia: Normoxia/Placebo v Normoxia/Nimodipine.
3. Effect of nimodipine in hypoxia: Hypoxia/Placebo v Hypoxia/Nimodipine.

Statistical differences between these conditions were assessed using permutation tests,^42^ with the FSL tool randomise for the voxelwise analyses, using 5000 permutations, and with the tool PALM (Permutation Analysis of Linear Models (https://fsl.fmrib.ox.ac.uk/fsl/fslwiki/PALM) for the region-based analyses, using 10,000 permutations followed by the approximation of the tail of the permutation distribution using a generalized Pareto distribution.^43^ Each *p-*value was adjusted for six comparisons, these being the three differences in the positive and negative directions. Significance for all tests was established at the level alpha < 0.05. Finally, a repeated measures ANOVA was applied to the region-based data to investigate the presence of an interaction between drug conditions and oxygenation conditions to investigate whether nimodipine significantly modulates the response to hypoxia.

For the voxelwise analyses in each modality, to account for the repeated measures, the differences were calculated between each contrast (e.g. normoxia/placebo v hypoxia/placebo) and then entered into a one sample *t-*test, assessed with sign flippings. Voxelwise analyses used threshold-free cluster enhancement (TFCE),^44^ with familywise error rate corrected across space. Results were considered significant at the level alpha < 0.05.

The Pearson correlation coefficient was used to investigate correlations between blood levels of the drug nimodipine and MRI changes seen between the Hypoxia/Placebo and Hypoxia/Nimodipine conditions with results considered significant at *p* < 0.05.

## Results

Complete laboratory and MRI datasets were obtained in 18 out of the 20 subjects. Two subjects withdrew from the study due to scheduling issues preventing completion of all sessions. During pre-processing and prior to unblinding, significant motion artifact was discovered in one VEPCASL dataset throughout all sessions. This subject was therefore excluded leaving only 17 subjects for the VEPCASL group analysis.

### Physiological data

**Figure 2.**
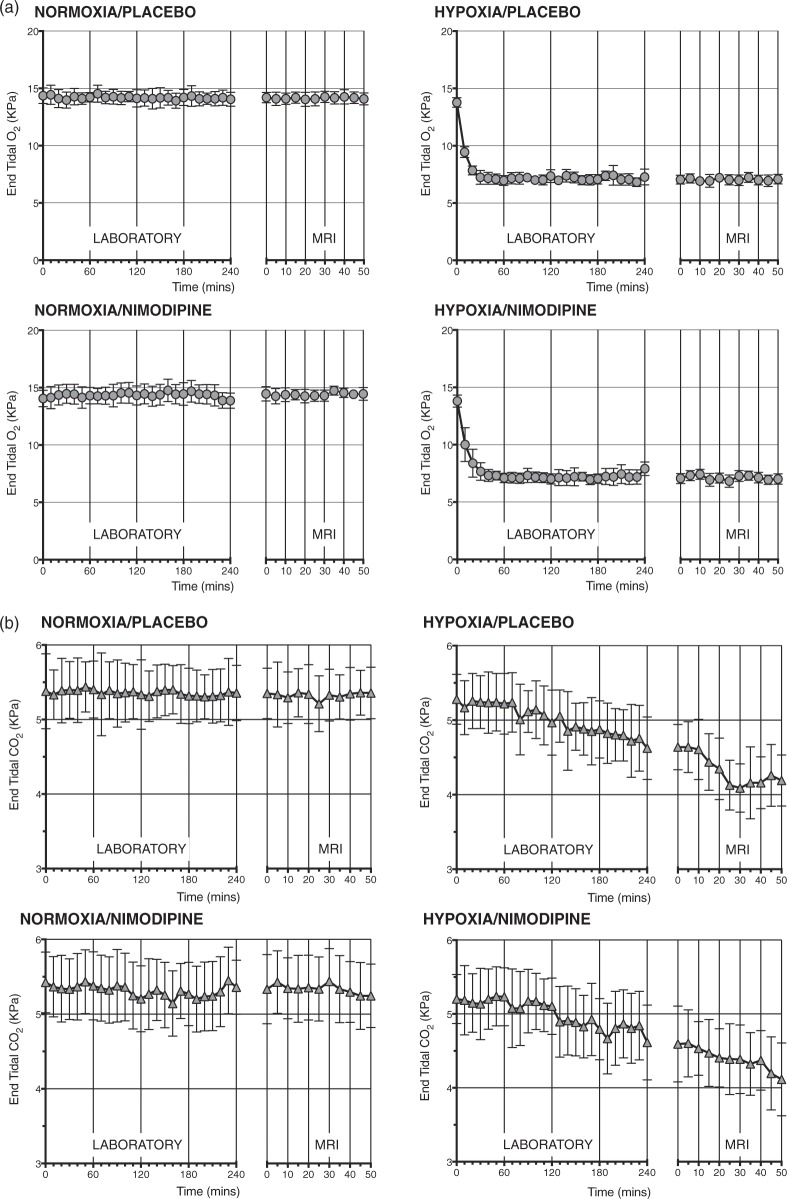
(a) PetO_2_ values from the laboratory and MRI sessions. (b) PetCO_2_ values from the laboratory and MRI sessions.

Figure 2(a) shows changes in mean PetO_2_ and Figure 2(b) shows changes in end tidal carbon dioxide (PETCO_2_) levels throughout the laboratory and MRI sessions for all participants. The differences in PetCO_2_, PETCO_2_, SpO_2_, heart rate and non-invasive blood pressure between each condition are summarized in Table 1 along with significance values.

**Table 1.** Physiological data, nimodipine levels and phase contrast MRI results for each experimental condition.

	Normoxia placebo	Normoxia nimodipine	Hypoxia placebo	Hypoxia nimodipine
SpO2 (%)	98 ± 1***	98 ± 1	85 ± 6***	84 ± 7
HR (bpm)	67 ± 2***	68 ± 2	77 ± 3***	79 ± 3
SBP (mmHg)	115 ± 15	114 ± 12	119 ± 12	116 ± 16
DBP (mmHg)	64 ± 6	62 ± 7	64 ± 6	64 ± 6
MAP (mmHg)	80 ± 7	79 ± 9	82 ± 9	82 ± 8
PETO2 (KPa)	14.17 ± 0.12***	14.36 ± 0.19	7.38 ± 1.18***	7.48 ± 1.21
PETCO2 (KPa)	5.35 ± 0.04***	5.32 ± 0.08	4.80 ± 0.37***	4.83 ± 0.29
Nimodipine levels (ng/ml)	0	18.90 ± 20.33	0	24.07 ± 24.62
Phase contrast Velocities				
Left carotid (cm/s)	30.93*** ± 5.32	28.66 ± 5.92	28.37*** ± 4.97	27.07 ± 4.46
Right carotid (cm/s)	31.22* ± 4.19	31.05 ± 5.67	30.38* ± 5.21	28.24 ± 5.03
Left vertebral (cm/s)	14.50 ± 3.55	14.28 ± 2.39	13.87 ± 3.43	13.22 ± 3.10
Right vertebral (cm/s)	11.95 ± 3.59	11.92 ± 4.43	11.96 ± 4.75	11.51 ± 2.99

*** = *p* < 0.001.

Compared with normoxia, as expected, the hypoxia condition significantly reduced the PetO_2_ in both the placebo and nimodipine conditions. There was a drop in PETCO_2_ values for both the placebo and nimodipine conditions with hypoxia as the laboratory and MRI sessions progressed.

Nimodipine had no significant effect on either PetO_2_ or PETCO_2_ compared to placebo in either the normoxia or hypoxia conditions. Hypoxia resulted in a significant drop in SpO_2_ and increase in heart rate in both placebo and nimodipine conditions. There was no significant difference in mean arterial pressure between normoxia and hypoxia and with nimodipine.

### Nimodipine levels

Serum nimodipine levels for each experimental condition are presented in the Table 1. Importantly, there was no significant difference in nimodipine levels between the normoxia and hypoxia sessions (18.90 ±20.33 ng/ml v 24.07 ± 24.62 ng/ml, *p* = 0.73).

### MRI results

**Figure 3.**
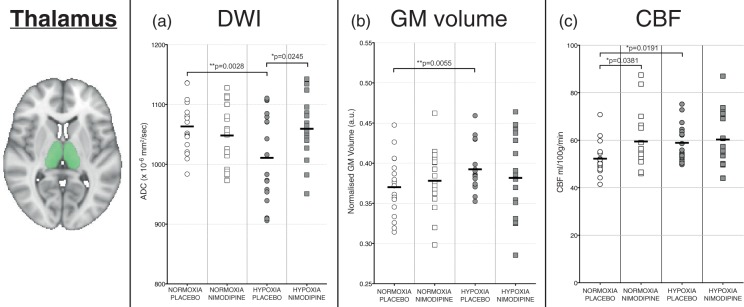
Graphs showing changes in the thalamus for (a) apparent diffusion coefficient (ADC), (b) volume and (c) cerebral blood flow in each of the four experimental conditions. The area shaded in green represents the region of interest mask of the thalamus used.

**Figure 4.**
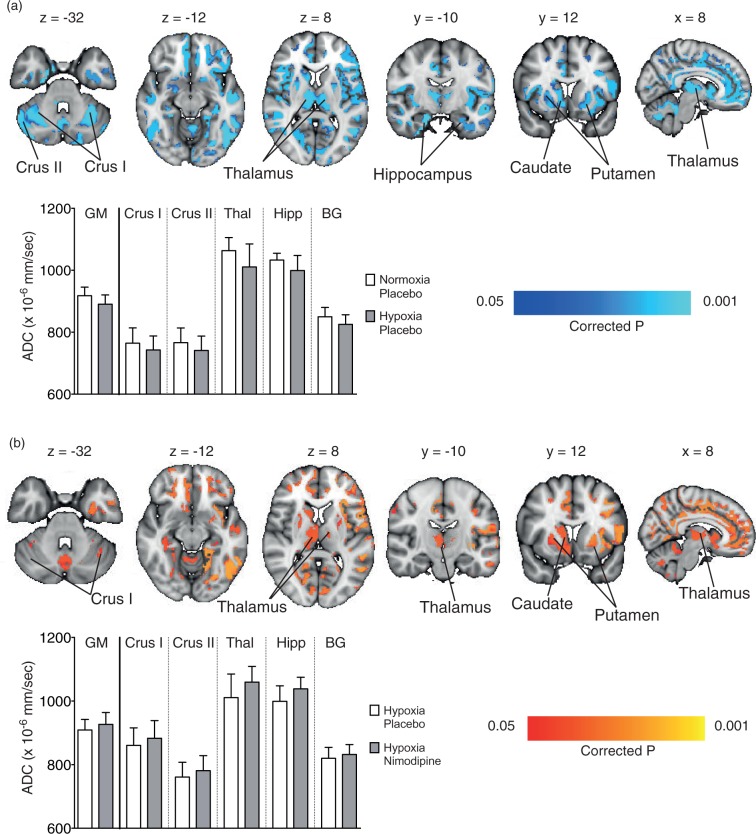
(a) Regional areas of decreased grey matter apparent diffusion coefficient (ADC) in the hypoxia/placebo condition compared to normoxia/placebo. (In yellow, *p* < 0.05 FWE-corrected for multiple comparisons, overlaid onto an MNI152 standard template). (b) Regional areas of increased grey matter apparent diffusion coefficient (ADC) in the hypoxia/nimodipine condition compared to hypoxia/placebo. (In yellow, *p* < 0.05, overlaid onto an MNI152 standard template. Crus I and Crus II = crura of cerebellum, BG = basal ganglia.

**Figure 5.**
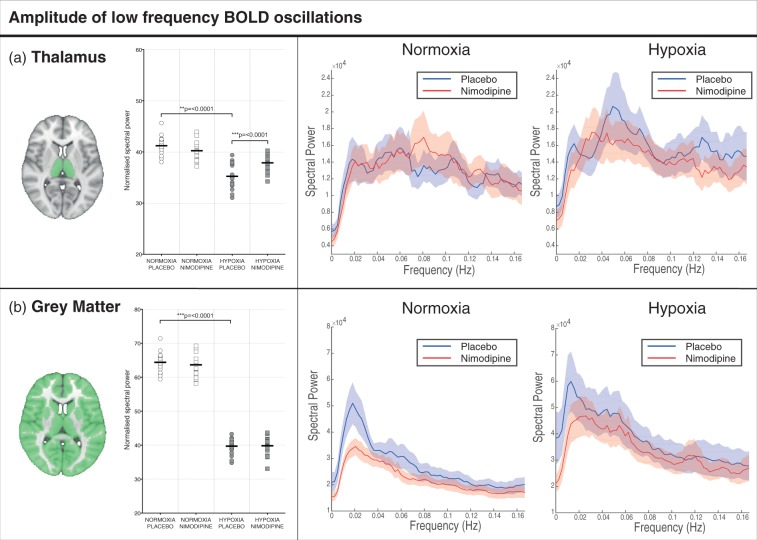
(a)Thalamus and (b) whole brain grey matter change in ALFF and frequency spectra for placebo and nimodipine conditions in normoxia and hypoxia (graphs show mean (solid line) with 95% CI (shaded areas)). The area shaded green represents the region of interest mask used.

**Figure 6.**
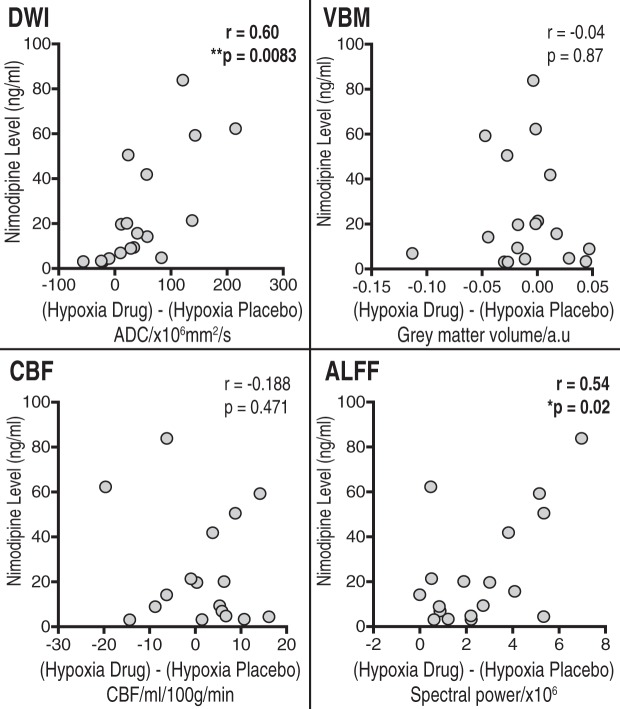
Correlations for the VBM, DWI, CBF and normalised ALFF analysis between nimodipine levels and effect of nimodipine in hypoxia (hypoxia/drug – hypoxia/placebo).

Figure 3 shows the MRI results for the thalamus from the study for the DWI (Figure 3(a)), structural (Figure 3(b)) and perfusion-weighted images (Figure 3(c)). Figure 4 shows data from the voxelwise whole-brain comparisons for the DWI data. Figure 5 shows the results of the ALFF analysis. Figure 6 shows the correlations between nimodipine levels and change in MRI data for Hypoxia/Placebo and Hypoxia/Nimodipine conditions.

Tables S2 and S3 in the supplementary material show the common local peaks of the significant clusters for Figure 5.

#### Effect of acute hypoxia

##### Diffusion-weighted imaging

There was a significant decrease in ADC values in the thalamus for the hypoxia condition compared to the normoxia condition (1061 ± 40 vs. 1011 ± 75 × 10^−6^mm^2^/s, *p* = 0.0002) (Figure 3(a)). Whole brain voxelwise analysis confirmed regional variation in GM ADC associated with the hypoxia condition, with especially noticeable decreases in ADC seen in the subcortical structures (thalamus, basal ganglia, hippocampus) as well as the cerebellum (crus I and II regions – Figure 4(a)).

##### T1-weighted imaging

Figure 3(b) shows that hypoxia resulted in a significant apparent increase in GM volume in the thalamus ROI when compared to normoxia (0.3924 ± 0.028 vs. 0.370 ± 0.037 a.u., *p* = 0.0027). Looking voxelwise over the whole brain though, there was no significant difference in GM volume.

##### Perfusion-weighted imaging

Figure 3(c) shows the results from the ASL perfusion-weighted imaging data for each experimental condition. There was an increase in CBF in the thalamus ROI for the hypoxia condition compared to normoxia (59.15 ± 10.00 vs. 50.34 ±6.68 ml/100 g/min, *p* = 0.0191). However, looking at the whole brain GM voxelwise analysis, there were no areas of significant difference in GM CBF between the normoxia and hypoxia conditions.

Table 1 also presents the results for the phase contrast imaging for each experimental condition. Flow through both carotid arteries was significantly higher in the normoxia condition when compared to hypoxia. This was not the case for the vertebral arteries, where there was no significant difference in flow values.

##### Amplitude of low frequency fluctuations

There was a large increase in normalised ALFF in the thalamus (35.24 ± 2.43 vs. 41.20 ± 1.85, *p* = <0.0001) with hypoxia compared to normoxia (Figure 5). Results for GM are provided for comparison along with full power spectra for both the thalamus ROI and whole brain GM. These differences remained significant when the additional data physiological denoising was conducted as described in the materials and methods section (See Supplementary Data).

#### Effect of nimodipine under conditions of normoxia

##### Diffusion-weighted imaging

There was no significant difference between nimodipine and placebo in the thalamus (1061 ± 40 vs. 1050 ± 55 × 10^−6^ mm^2^/s, *p* = 0.1363). There was also no significant regional or global effect on whole brain GM ADC values using voxelwise analysis.

##### T1-weighted imaging

Like the DWI data, compared with placebo, there was no significant difference in volume in the nimodipine condition for the thalamus (0.370 ± 0.037 vs. 0.3781 ± 0.035 a.u., *p* = 0.2370) and no significant difference seen over whole brain GM using voxelwise analysis.

##### Perfusion-weighed imaging

There was a significantly increased thalamic CBF in the nimodipine condition compared with placebo (59.49 ± 11.70 ml/100 g/min vs. 52.26 ± 6.91 ml/100 g/min, *p* = 0.037). However, there was no significant difference seen over whole brain GM using voxelwise analysis. Looking at the phase contrast results, velocities through carotid and vertebral arteries were not significantly different between the nimodipine and placebo conditions.

##### Amplitude of low frequency fluctuations

There was a significant reduction in normalised ALFF in the thalamus between nimodipine and placebo in the normoxia condition (41.20 ± 1.85 vs. 40.24 ± 1.88, *p* = 0.028). Again, full power spectra and values for GM are provided for comparison in Figure 5.

#### Effect of nimodipine under conditions of hypoxia

##### Diffusion-weighted imaging

In the hypoxia condition, there was a significant increase in ADC values with nimodipine towards baseline in the thalamus compared to placebo (1059 ± 49 vs. 1048 ± 34 × 10^−6^mm^2^/s, *p* = 0.0171). There was also a significant interaction effect between nimodipine and hypoxia (*p* = 0.0002). Furthermore, there was also a significant positive correlation between the change in ADC values in the thalamus between hypoxia/placebo and hypoxia/drug conditions and serum nimodipine levels (*r* = 0.60 *p* = 0.0083) with higher nimodipine levels in subjects associated with greater change in ADC values (Figure 6).

Figure 4(b) shows the voxelwise changes between the hypoxia/placebo and hypoxia/nimodipine conditions. Again, there was a significant increase in ADC across multiple subcortical regions including the thalamus, basal ganglia (putamen and caudate), hippocampus and cerebellum with the administration of nimodipine.

Finally, voxelwise comparison was performed between normoxia/placebo and hypoxia/nimodipine which showed no significant differences over whole brain GM confirming the reversal of the effect of acute hypoxia.

##### T1-weighted imaging

Nimodipine had no significant effect on the volume of the thalamus (0.382 ± 0.050 vs. 0.392 ± 0.028 a.u., *p* = 0.5291) in hypoxia versus placebo. There was no significant interaction effect either between drug condition and oxygenation condition (*p* = 0.1480). Voxelwise analysis showed no significant effect on whole brain GM volumes.

Finally, there was no significant correlation between the change in the thalamus volume between hypoxia/placebo and hypoxia/drug conditions and serum nimodipine levels (r = − 0.04, *p* = 0.87).

##### Perfusion-weighted ASL imaging

We did not observe any significant difference in the thalamus CBF under conditions of hypoxia for nimodipine versus placebo (60.29 ± 12.00 vs. 58.48 ± 7.86 ml/100 g/min, *p* = 0.9158) or any voxelwise differences in GM CBF. There was also no significant interaction effect (*p* = 0.4042). There was also no significant correlation between the change in the thalamus CBF between hypoxia/placebo and hypoxia/drug conditions and serum nimodipine levels (r = −0.188 *p* = 0.4710).

Looking at the phase contrast results, velocities through both carotid and vertebral arteries were not significantly different between the nimodipine and placebo conditions under conditions of hypoxia.

##### Amplitude of low frequency fluctuations

In the thalamus, there was a significant increase in ALFF with nimodipine when compared to placebo (37.87 ± 1.66 vs. 35.24 ± 2.43, *p* = 0.0003)(Figure 5). Furthermore, there was a significant interaction effect between drug and oxygenation conditions (*p* = 0.0002). Like the DWI results, the degree of increase in thalamic ALFF with nimodipine in hypoxia showed a significant positive correlation with nimodipine levels (r = 0.54, *p* = 0.02).

## Discussion

The aim of this study was to test the hypothesis that calcium channel activity mediates the cerebral response to inspiratory hypoxia in vivo in humans. Through this, we aimed to clarify the role of calcium in the pathophysiology of acute brain injury in clinical practice. Several notable findings have arisen from this study. Firstly, acute hypoxia causes a clear reduction in ADC and ALFF values in the thalamus. These changes are accompanied by an apparent increase in thalamic volume, together with an increase in thalamic CBF. Calcium channel blockade with nimodipine attenuated the effects of cerebral hypoxia on ADC and ALFF values seen in the thalamus and other cortical/sub-cortical areas, returning values back towards baseline levels seen in normoxia. Finally, changes in ADC and ALFF with nimodipine showed a significant correlation with nimodipine levels in hypoxia.

### Cerebral effects of acute hypoxia

Previous studies using MRI to investigate the effect of acute hypoxia on the brain have reported contradictory results showing either increased^17,19^ or reduced^18,45,46^ ADC values. This may be due to differences in study/MRI protocols, duration of hypoxic stimulus and barometric pressures. In our study, the duration of hypoxia was chosen at 4 h in order to compensate for initial changes in CBF and ventilation that occur in the first 30 min post-hypoxic stimulus^47^ and to allow potential MRI biomarkers of pathophysiological changes associated with hypoxia to develop. Our results demonstrate that the thalamus is sensitive to acute inspiratory hypoxia with significantly decreased ADC values. These results were replicated in a secondary voxelwise whole brain analysis. This further highlighted decreased ADC in other subcortical structures such as the caudate, putamen, hippocampus and cerebellum – areas also known to be susceptible to hypoxic-ischaemic damage. Reduced ADC values are generally interpreted to be due to cytotoxic oedema associated with intracellular swelling, and are associated with changes in cellular energy status responsible for reduced Na + /K + ATPase pump and altered ionic homeostasis.^17,19^

Analysis of the T1-weighted data shows that thalamic volume was apparently increased with acute hypoxia. Other studies have reported similar effects following exposure to acute hypoxia with increased total brain and GM volumes associated with reduced whole brain ADC.^18,45^ This increase is likely to result from cellular swelling due to cerebral oedema associated with inspiratory hypoxia. MRI evidence of cerebral oedema has also been noted as early as the first hour of hypoxic exposure, associated with significant shifts in intracranial CSF volumes.^48^

The results from the VEPCASL analysis suggest this increase may be due to the increase in CBF seen with acute hypoxia on CBF in the thalamus. Voxelwise changes in CBF were not seen over the whole-brain which is in concordance with other studies which have reported no difference in whole brain GM CBF in acute poikilopcapnic hypoxia.^45^ It may also reflect the cerebral vasoconstriction that occurs because of the increased ventilatory response to hypoxia as well as issues with signal-to-noise ratio seen with ASL studies.

### Effects of calcium channel blockade on cerebral physiology during normoxia

In normoxia, nimodipine had no significant effect on ADC nor GM volume in the thalamus. However, there was a significant 14% increase in thalamic CBF and a reduction in ALFF. Nimodipine is characterized by a highly selective action on cerebral blood vessels and a high affinity to receptors in the cerebral cortex – hence the interest in its use clinically following acute brain injury. However, the literature investigating the effect of nimodipine on CBF is inconsistent. Early studies in animals under conditions of ischaemia demonstrated no effect on cerebral metabolism but increases in CBF due to a direct vasodilatory effect across all brain regions, particularly following disruption of the blood–brain barrier.^4,49–51^ However, nimodipine had no significant effect on normal CBF or metabolism in animals.^52^ Early studies into nimodipine in both healthy volunteers and patients suffering from stroke suggested a small overall increase in CBF after nimodipine treatment, with a redistribution of blood to hypoperfused regions in patients.^53,54^ However, subsequent studies have shown no effect of nimodipine in healthy volunteers in concordance with the results of our study. ^55^ This may be as a result of differences in drug dose and timing as well as differences in CBF measurement techniques.

### The effect of nimodipine under conditions of cerebral hypoxia

Results from this study demonstrate that nimodipine reverses decreases in both ADC and ALFF seen in the thalamus with acute hypoxia without an apparent effect on CBF The whole brain analysis also showed similar increases in ADC across the GM including subcortical structures such as the putamen, caudate, hippocampus and cerebellum. There was also a significant positive correlation between the change in ADC values between Hypoxia/Placebo and Hypoxia/Nimodipine conditions and serum nimodipine levels in the thalamus. Voltage-gated calcium channels are essential for calcium signaling in excitable cells and play a key role in the final common pathway of acute cerebral ischaemia. Furthermore, L-type channels are known to display a particularly high sensitivity to hypoxia.^56^ In experimental models of focal and global ischemia, L-type calcium channel antagonism with nimodipine has been shown to have a neuroprotective effect, attenuating cognitive dysfunction and increasing the hypoxic tolerance of brain tissue.^57,58^ Nimodipine has also consistently been shown to reduce the incidence of secondary cerebral ischaemia and improve clinical outcomes after aneurysmal subarachnoid haemorrhage^59–61^ – without any effect on cerebral vasodilatation.

Our results suggest that calcium channel blockade with nimodipine acts to reduce cytotoxic cerebral oedema following acute cerebral hypoxia without increased CBF. Other studies have suggested that nimodipine may have an effect on cerebral metabolism^62^ which may underlie this effect on ADC and explain the clinical benefit seen in SAH, a condition characterized by high levels of cytotoxic cerebral oedema. Finally, there did not appear to be a difference in PetCO_2_ with nimodipine, suggesting that changes in CBF are unlikely to be due to differences in ventilatory control. Other studies have demonstrated that nimodipine does not seem to exert a significant impact on the effect of hyperventilation in reducing regional CBF^63^ and interferes with the CBF response to alterations in arterial CO2.^64^ This may partially explain why there was no significant increase in CBF with nimodipine under conditions of hypoxia.

The variation in nimodipine levels despite consistent dosing between subjects suggests that the pharmacodynamics of the oral administration route may be an important factor in the use of nimodipine clinically in patients. There was also a strong correlation between serum nimodipine levels and changes in ADC and ALFF seen with nimodipine in hypoxia. This highlights that monitoring of drug levels may be important if consistent clinical outcome benefits are to be achieved, e.g. following subarachnoid haemorrhage.

### ALFF

To date, no other studies have considered the exact role of the ALFF in cerebral hypoxia. Clinical studies have suggested that changes in ALFF in disease states may be a marker of brain damage. Altered ALFF in frontal brain regions is seen in patients recovering from traumatic brain injury,^20^ as well as in the cerebellum in patients with migraine and depression.^65,66^ Increased ALFF in the thalamus is also positively correlated with microstructural damage seen on MRI in multiple sclerosis.^67^ Our results show that acute inspiratory hypoxia results in a significant drop in thalamic ALFF in the BOLD signal replicating changes seen in disease states. Calcium channel blockade with nimodipine in normoxia also leads to a significant decrease in ALFF in the thalamus – potentially due to the role of calcium oscillations in resting brain neuronal activity. However, in the presence of hypoxia, the administration of nimodipine significantly increases ALFF normalizing levels to those seen in normoxia. Regional heterogeneity in the BOLD response to hypoxia has previously been reported in both animal studies^68,69^ and studies in humans.^70^ The underlying mechanisms that could lead to this are poorly understood and include different changes in total deoxyhaemoglobin for a fixed change in arterial oxygen saturation due to different blood volumes and/or different oxygen extraction fractions and flow-induced variation in saturation and signal intensity.^69^ It may be that subcortical structures such as the thalamus have a different oxygen extraction fraction at rest than other areas of the brain and are therefore more sensitive to changes in arterial oxygen content. Though the absence of significant correlation between CBF and change in ALFF with nimodipine in hypoxia suggests that this difference may be related to a mechanism of action of calcium channel blockade on neurons (potentially via an effect on low-frequency oscillations in calcium levels) rather than a direct vascular effect.

## Conclusion

In summary, we have provided evidence for a preferential effect of calcium channel blockade with nimodipine under conditions of acute cerebral hypoxia in reversing changes in ADC and ALFF in the thalamus. These changes to ADC and ALFF may underlie the benefit seen clinically in conditions like SAH where significant global cytotoxic cerebral oedema is seen with early brain injury. Further research is needed into the importance of changes in ALFF with hypoxia and whether this represents a biomarker of cerebral tissue hypoxia in acute acquired brain injury.

## Supplementary Material

Supplementary material
