# Supplementary material for: Calcium channel blockade with nimodipine reverses MRI evidence of cerebral oedema following acute hypoxia
Source: J Cereb Blood Flow Metab. 2017 Aug 31;39(2):285–301. doi: 10.1177/0271678X17726624 (PMC6360646; doi:10.1177/0271678X17726624)

SUPPLEMENTARY MATERIAL

Table S1: Common local peaks of the significant clusters (P < 0.05 FWE-corrected) showing reduced ADC in hypoxia in contrast to normoxia in the placebo condition. Local maximum t values correspond to Figure 5A.

**Cortical Region**	**Side**	**MNI Coordinates**			**Local maximum t**
GM Visual Cortex V5	Lt	-52	-66	-6	6.6
GM Visual Cortex V2	Lt	-4	-70	30	3.98
GM Visual Cortex V1	Rt	18	-68	8	4.94
Auditory cortex	Rt	48	-16	8	4.78
Parahippocampal gyrus	Rt	20	-10	-30	5.66
Entorhinal cortex	Rt	22	-6	-36	5.47
Superior temporal gyrus	Rt	50	-22	0	5.93
Middle temporal gyrus	Rt	56	-20	-18	6.3
Inferior temporal gyrus	Rt	54	-52	-20	5.22
Temporal fusiform cortex	Lt	-32	-24	-24	4.26
Frontal operculum cortex	Rt	-42	28	2	5.63
Central operculum cortex	Rt	56	-8	14	4.0
Frontal pole	Lt	-46	48	0	4.42
Frontal medial cortex	Lt	-12	38	-10	3.37
Frontal orbital cortex	Lt	-48	28	-14	3.03
Inferior frontal gyrus	Lt	-54	14	16	5.18
Precentral gyrus	Lt	-12	-28	44	5.12
Postcentral gyrus	Lt	-62	-16	24	3.91
Paracingulate gyrus	Rt	8	48	-2	4.85
Cingulate gyrus	Rt	4	-48	20	4.44
Superior parietal lobule	Lt	-2	-38	58	3.52
Inferior parietal lobule	Rt	50	-34	30	4.95
Inferior parietal lobule (angular gyrus)	Rt	52	-52	18	4.3
Insular cortex	Lt	-42	4	-4	4.63
Precuneus cortex	Rt	22	-56	20	4.01
Thalamus	Rt	12	-18	8	5.0
	Lt	-16	-20	12	4.25
Putamen	Rt	32	-16	-2	4.74
Putamen	Lt	-28	-18	6	3.25
Caudate	Rt	10	6	4	3.13
Hippocampus	Lt	-22	-38	0	4.76
Amygdala	Lt	-26	-6	-20	3.4
**Cerebellum**					
Cerebellum (VIIb)	Lt	-22	-72	-46	6.15
Cerebellum (VIIb)	Rt	16	-74	-44	3.29
Cerebellum (Vermis VIIa)	Rt	6	-68	-38	6.4
Cerebellum (VI)	Lt	-12	-74	-20	6.4
Cerebellum (VI)	Rt	14	-64	-22	3.9
Cerebellum (Crus II)	Lt	-36	-62	-42	5.16
Cerebellum (Crus II)	Rt	48	-64	-48	4.12
Cerebellum (Crus I)	Rt	40	-66	-28	4.95
Cerebellum (Crus I)	Lt	-38	-56	-28	4.83
Cerebellum (I-IV)	Rt	8	-50	-20	4.39
Cerebellum (I-IV)	Lt	-4	-54	-18	3.94

Table S2: Common local peaks of the significant clusters (P < 0.05 FWE-corrected) showing increased ADC with nimodipine in hypoxia in contrast to placebo. Local maximum t values correspond to Fig. 5B. GM = Grey matter

**Cortical Region**	**Side**	**MNI Coordinates**			**Local maximum t**
GM Visual Cortex V2	Lt	-18	-54	-6	3.05
GM Visual Cortex V1 (BA 17)	Lt	-2	-64	4	3.18
Superior parietal lobule	Lt	-26	-66	48	4.04
Supramarginal gyrus	Lt	-56	-48	24	4.07
Inferior parietal lobule	Rt	58	-36	52	6.3
Lateral occipital cortex	Lt	-42	-74	-6	5.33
Frontal pole	Rt	24	56	-6	4.02
Frontal pole	Lt	-38	50	-2	4.08
Premotor cortex (BA 6)	Rt	8	2	48	3.36
Entorhinal cortex	Lt	-30	-8	-42	5.76
Precentral gyrus (BA 44)	Lt	-52	6	8	3.45
Postcentral gyrus	Lt	-68	-14	14	6.11
Postcentral gyrus	Rt	66	-8	32	3.06
Superior frontal gyrus (BA 1)	Lt	-20	36	36	4.21
Middle frontal gyrus (BA44)	Rt	26	32	38	4.77
Superior temporal gyrus	Lt	-56	-38	8	3.34
Middle temporal gyrus	Lt	-58	-46	-4	4.03
Middle temporal gyrus	Lt	-44	-28	-26	3.21
Temporal pole	Lt	-34	6	-38	4.22
Cingulate gyrus	Rt	10	-50	24	3.95
Paracingulate gyrus	Rt	10	46	-4	3.8
Hippocampus	Rt	22	-8	-24	4.19
Parahippocampal gyrus	Lt	-24	-40	-16	3.31
Parahippocampal gyrus	Rt	34	-34	-14	3.36
Thalamus	Rt	8	-16	0	3.59
Hippocampus	Lt	-18	-38	2	3.94
Putamen	Lt	-30	0	-6	3.47
Putamen	Rt	24	8	4	4.15
Subcallosal cortex	Rt	12	16	-14	3.08
Insular cortex	Lt	-42	-12	2	3.04
**Cerebellum**					
Cerebellum (Crus II)	Lt	-18	-78	-48	4.08
Cerebellum (VI)	Lt	-22	-72	-22	3.68
Cerebellum (VI)	Rt	18	-62	-24	4.21
Cerebellum (V)	Rt	16	-50	-24	4.11
Cerebellum (I-IV)	Lt	-2	-52	-14	3.28

Figure S3: ALFF results in the thalamus for each experimental condition following supplementary data denoising with FIX and RETROICOR

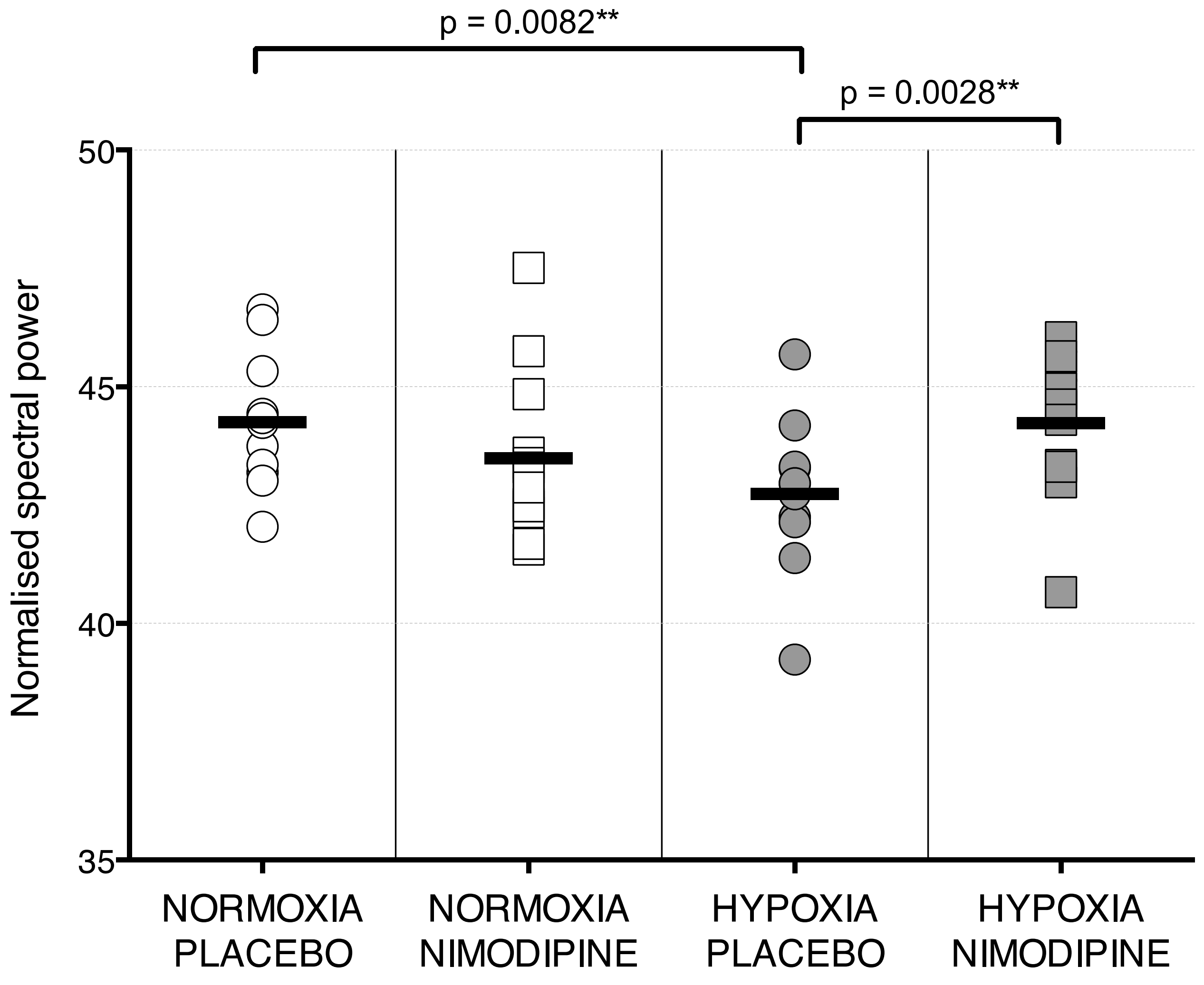

Figure S4: Graphs of spectral power for each experimental condition for the thalamus ROI following denoising with both ICA and RETROICOR

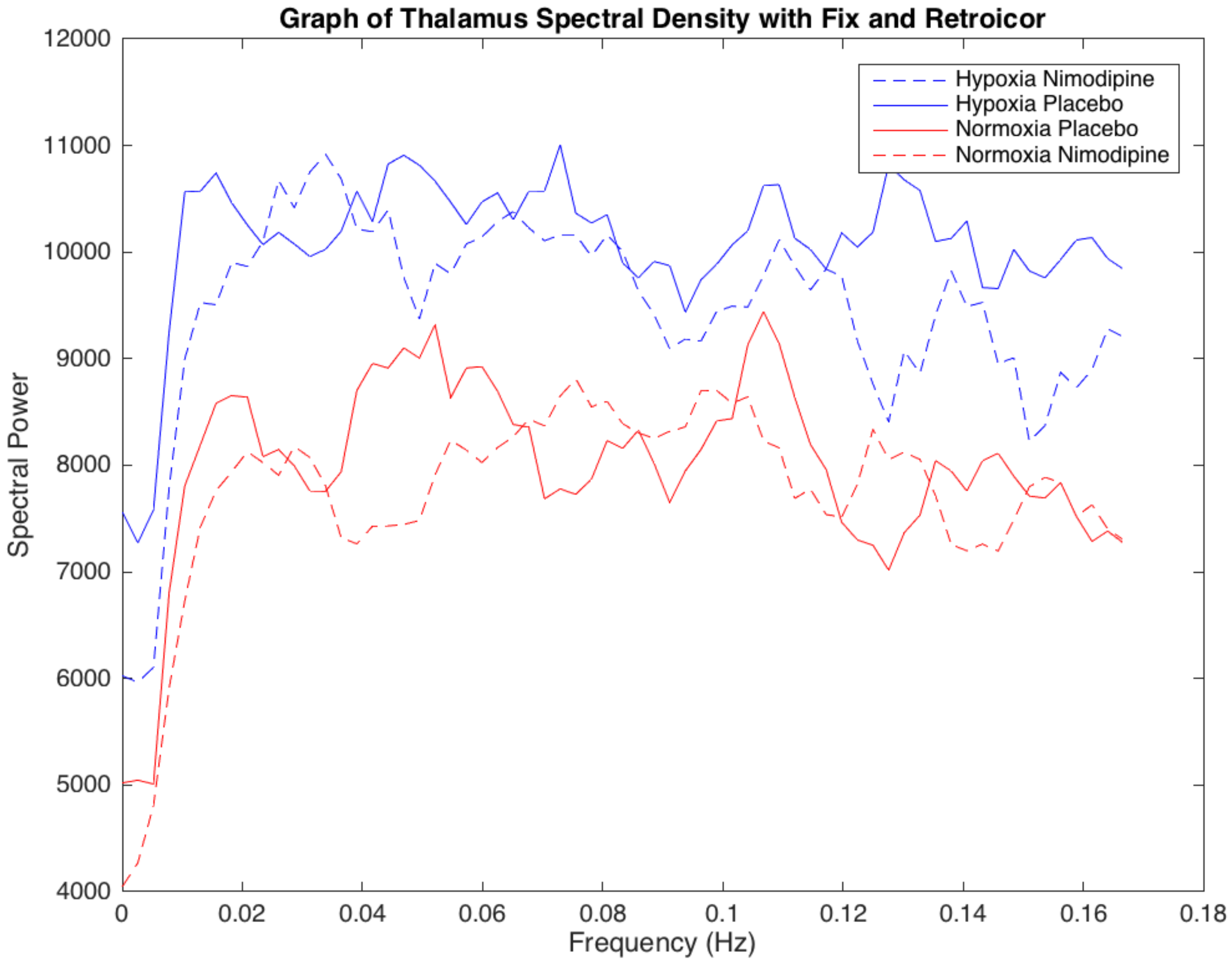

Figure S5: Graphs of spectral power for each experimental condition for whole brain grey matter following denoising with both ICA and RETROICOR

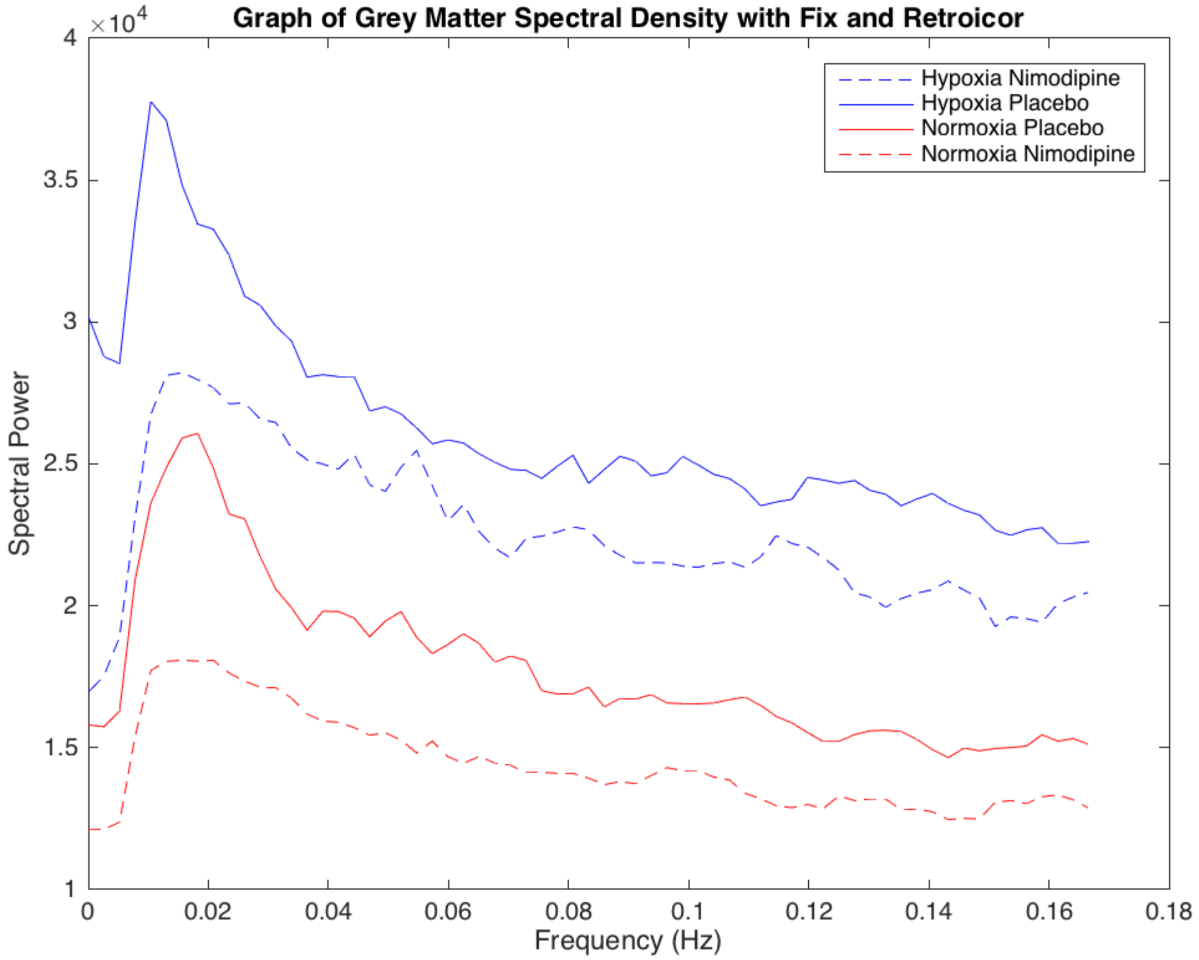

Figure S6: Graphs of spectral power for each experimental condition for the thalamus ROI with no data denoising

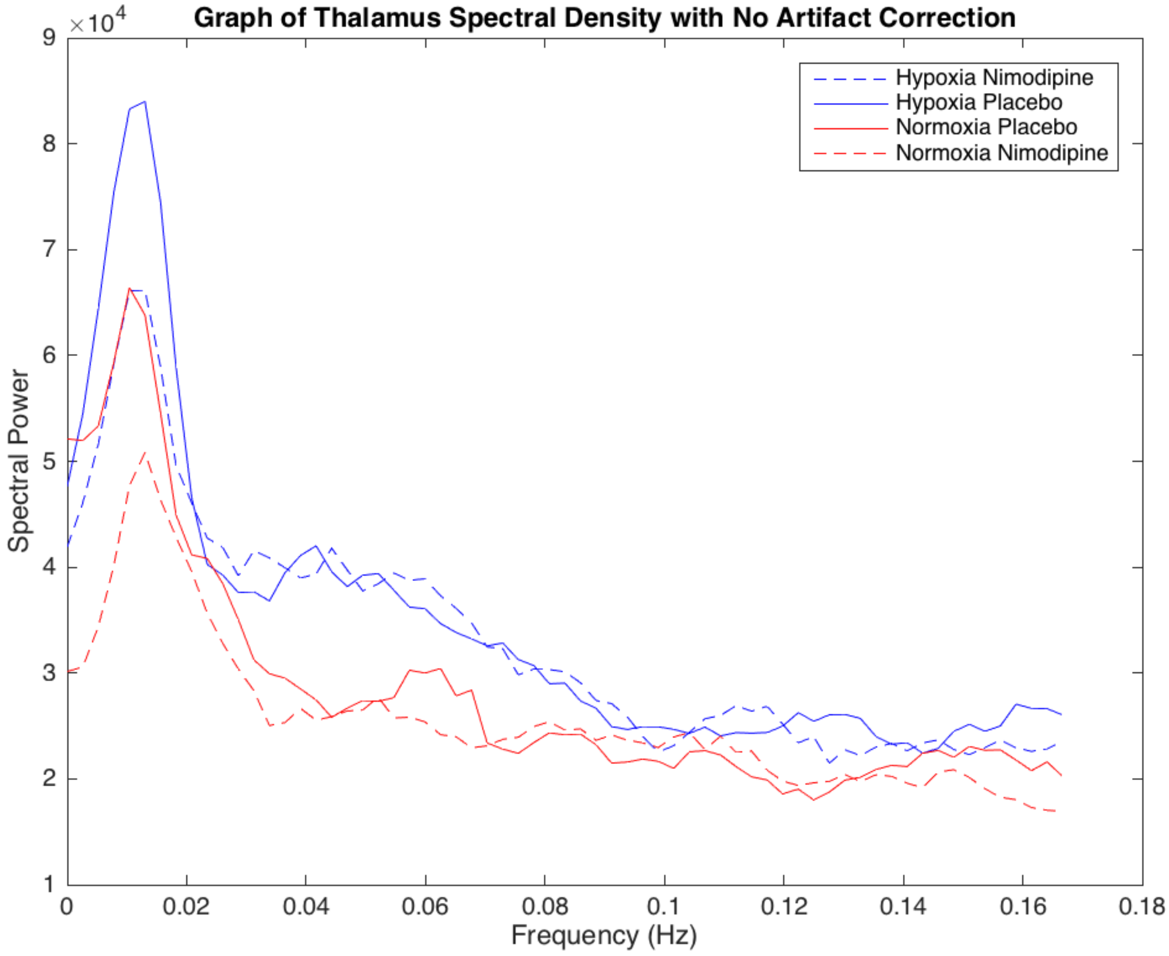

Supplement: Supplementary material [file supplementary_material4.pdf]
